# A20 as a Potential New Tool in Predicting Recurrence and Patient’s Survival in Oral Squamous Cell Carcinoma

**DOI:** 10.3390/cancers15030675

**Published:** 2023-01-21

**Authors:** Steffen Spoerl, Ramona Erber, Michael Gerken, Juergen Taxis, Nils Ludwig, Felix Nieberle, Niklas Biermann, Carol Immanuel Geppert, Tobias Ettl, Arndt Hartmann, Philipp Beckhove, Torsten E. Reichert, Gerrit Spanier, Silvia Spoerl

**Affiliations:** 1 Department of Cranio-Maxillofacial Surgery, University Hospital Regensburg, 93053 Regensburg, Germany; 2 Institute of Pathology, University Hospital Erlangen, Friedrich-Alexander-Universität Erlangen-Nürnberg (FAU), Comprehensive Cancer Center Erlangen-EMN, 91054 Erlangen, Germany; 3 Tumor Center, Institute for Quality Management and Health Services Research, University of Regensburg, 93053 Regensburg, Germany; 4 Department of Plastic, Hand and Reconstructive Surgery, University Hospital Regensburg, 93053 Regensburg, Germany; 5 Division of Interventional Immunology, Leibniz Institute for Immunotherapy, 93053 Regensburg, Germany; 6 Department of Internal Medicine 5—Haematology and Oncology, University Hospital Erlangen, Friedrich-Alexander-University Erlangen-Nürnberg (FAU), 91054 Erlangen, Germany

**Keywords:** oral squamous cell carcinoma, OSCC, A20, tissue microarray, TNFAIP3

## Abstract

**Simple Summary:**

Here, we examined the presence and clinical relevance of A20 in oral squamous cell carcinoma (OSCC). Using a tissue microarray (TMA), immunohistochemical A20 expression as well as tumor-infiltrating lymphocytes (TIL) were analyzed. Hereby, A20 expression was significantly increased in TILs and patients with elevated A20 expression in stromal CD3+ cells showed ameliorated survival in uni- as well as multivariable analyses.

**Abstract:**

A20, known as a potent inhibitor of NF-κB signaling, has been characterized in numerous clinical as well as preclinical studies. Recently, especially in various malignant diseases, the prognostic and therapeutic relevance of A20 was investigated. In oral squamous cell carcinoma (OSCC) however, the characterization of A20 is uncharted territory. We analyzed a tissue microarray (TMA) of 229 surgically-treated OSCC patients (2003–2013). Immunohistochemical (IHC) stainings were performed for A20 and CD3; additionally, standard haematoxylin-eosin staining was applied. IHC findings were correlated with a comprehensive dataset, comprising clinical and pathohistological information. A20 expression was analyzed in tumor cells as well as in tumor infiltrating lymphocytes (TILs) and correlated with the overall survival (OS) and recurrence-free survival (RFS) using uni- and multivariable Cox regression. The median follow-up time was 10.9 years and the A20 expression was significantly decreased in CD3+ TILs compared to mucosa-infiltrating lymphocytes (MILs). In the Kaplan–Meier analyses, higher A20 expression in TILs was correlated with better OS (*p* = 0.017) and RFS (*p* = 0.020). In the multivariable survival analysis, A20 overexpression correlated with improved OS (HR: 0.582; 95% CI 0.388–0.873, *p* = 0.009) and RFS (HR 0.605; 95% CI 0.411–0.889, *p* = 0.011). Our results indicate a novel prognostic role for A20 in OSCC. Due to its elevated expression in TILs, further research is highly desirable, which therefore could offer new therapeutic opportunities for patients suffering from OSCC.

## 1. Introduction

Since Rudolf Virchow associated inflammation with development of malignant diseases in 1863 [1], several inflammatory cascades in malignant diseases became illuminated [2]. Today, over 150 years after Virchow hypothesized that chronic inflammation might resemble an origin for cancer, the correlation between inflammation, immune response and cancer are broadly accepted [3].

With over 15% of malignancies arising on the soil of chronic inflammation [4], inflammation-induced tumors nowadays comprise a relevant clinical as well as public health topic. Mechanistically, a possible explanation for the development of cancer on the basis of chronic inflammation might simply be associated due to a normal host response [3], hereby, the generation of reactive oxygen and nitrogen species, which normally represents a physiological host response on viral and bacterial infections [3]. Consecutively, numerous endogenous cells undergo relevant DNA damage caused by free radicals and thereby possibly discover malignant degeneration. Apart from this hypothesis, the immune response resulting from tissue damage or infection causes various kinds of leucocytes to produce proinflammatory factors like Tumor Necrosis Factor (TNF)-α or interleukin (IL)-6 [5]. In solid malignancies however, multiple chemokines and cytokines could already be identified to affect tumor cell migration, cell death, and therapy resistance [6,7,8,9]. Although these cytokines and chemokines picture a manifold orchestra of secreted factors, a large proportion of them are comprised in NF-κB signaling, which undoubtedly plays a major role in immune relation and inflammation [10]. In physiological circumstances, NF-κB signaling is hereby tightly regulated. However, in malignant diseases, constitutive activation of the NF-κB pathway was shown [11,12], leading to the clinically-relevant question of how to possibly deactivate this explicit pathway. In this regard, A20, also known as tumor necrosis factor, alpha-induced protein 3 (TNFAIP3), occurs as a potent inhibitor of NF-κB signaling [13,14], by interfering with ubiquitin enzyme complexes [15]. In rheumatoid and hematologic diseases, the role of A20 in regulating immune homeostasis has been shown in vitro as well in various translational and clinical studies [16,17,18,19].

In solid malignancies, the role of A20 in tumorigenesis as well as its prognostic relevance for recurrence and patient’s outcome lived a shadow existence for a long time. Recently, the role of A20 in hepatocellular carcinoma (HCC) was revealed, attributing A20 not only a hepato-protective effect but particularly a role as a relevant tumor suppressor in HCC [20]. In other tumor entities, the anti-neoplastic effect of A20 remains unclear, or its expression was even associated with an unfavourable patient outcome, as recently demonstrated in breast cancer [21,22].

In oral squamous cell carcinoma, the potential role of A20 is uncharted territory. Especially due to its expression on CD4+ T cells and CD8+ T cells and the thereby associated role of A20 in the antitumor immune response [23], we predominantly focused on the A20 expression on CD3+ T cells and subsequent translational implications on OSCC patients.

## 2. Materials and Methods

### 2.1. Patient Cohort

The retrospective cohort study was based on 229 adult Caucasian patients who received treatment for primary OSCC at the Department of Oral and Maxillofacial Surgery, University Hospital Regensburg between the years 2003 and 2014. The inclusion criteria for the present study included tumor resection of the primary lesion to negative margins (R0) which was combined with cervical lymph node dissection based on clinical and radiological findings. Every participant was staged based on the 7th edition of the UICC (Union Internationale contre le Cancer) guidelines [24]. Survival and clinicopathological analysis were achieved retrospectively. To reflect comorbidities and age, the age-adjusted Charlson Comorbidity Index CCI) was calculated for every patient as previously described [25]. Patient data were hereby retrieved from digital as well as paper-based medical records. Regarding the application of adjuvant treatment regimes, the decision of a multidisciplinary tumor board indicated radiotherapy or chemo-radiotherapy. Patient’s clinical as well as pathohistological characteristics are summarized in Table 1.

### 2.2. Immunohistochemical (IHC) Staining

Tissue microarrays (TMAs) were assembled as previously described [26]. Briefly, TMAs contained formalin-fixed, paraffin-embedded human OSCC tissues and corresponding non-malignant mucosal tissues of 229 patients of an OSCC cohort. Each single patient is represented by three cores (tumor center, the peripheral invasion front and the adjacent normal tissue). To evaluate the percentage of CD3+ T cells expressing A20, first, TMAs were cut into 2 µm-thick serial sections and stained as follows: (1) hematoxylin and eosin (H&E), (2) CD3 IHC and (3) A20 IHC. For CD3 IHC staining, the protocol was performed on a Ventana Benchmark Ultra automated platform (Ventana Medical Systems, Inc., Oro Valley, AZ, USA) using the SP7 clone (rabbit; dilution 1:150; Zytomed Systems GmbH, Bargteheide, Germany) with an incubation time of 32 min at 37 °C. For A20 IHC, the staining protocol was manually performed using a mouse monoclonal anti-A20 antibody (dilution 1:50 clone A20(A-12) sc-166692; Santa Cruz Biotechnology Inc., Dallas, TX, USA) with an incubation time of 30 min at room temperature (RT), followed by DAB and counterstaining with hematoxylin. 

### 2.3. Image Analysis

The stained TMA slides were scanned using the Pannoramic 1000 scanner (3DHistech, Budapest, Hungary) and visualized using the CaseViewer software (Version 2.4; 3DHistech, Budapest, Hungary). Evaluation was performed by a board-certified pathologist (R.E.) blinded to clinicopathological and outcome data visualizing H&E, CD3 and A20 scans of each TMA core next to each other. First, H&E slides, stained according to the standard in-house protocol, were reviewed regarding the morphology and growth pattern of OSCC. For each case, the semiquantitative percentage of A20 expression within stromal CD3+ T cells was assessed (ranging from 0–100%). 

### 2.4. Statistical Analysis

For the statistical analysis, SPSS version 26 software (IBM Germany GmbH, Ehningen, Germany) was used. Correlation analysis between clinicopathological parameters and A20 expression were calculated based on the Pearson’s Chi square test. For correlation analyses regarding A20 expression, a median cut-off (2.5) was used. Univariable survival analysis for overall survival (OS) and recurrence-free survival (RFS) was calculated based on the Kaplan–Meier method. OS was defined as the time from diagnosis to death by any cause. Additionally, RFS was defined as the time from surgery to tumor recurrence or death, whichever occurred first. Median follow-up was calculated using the reverse Kaplan–Meier method. Statistical relevance between survival values was calculated using the log-rank test. For risk adjustment in survival analysis, multivariable Cox regression was applied. Hereby, the results were reported with hazard ratios (HRs) and 95% confidence intervals (CIs). Additionally, proportional hazards assumptions were evaluated using the method of Grambsch and Therneau [27].

Multivariable analysis was adjusted for age-adjusted Charlson comorbidity score, UICC stage, lymph vessel and vein invasion, which proved to have *p* < 0.100 in univariable analysis. Tumor size, cervical node status and extranodal spread were dismissed in favor of UICC stage. All reported *p*-values are two-sided. Statistical significance was assumed if *p* < 0.05.

### 2.5. Single-Cell RNA-Seq Analysis

The Tumor Immune Single Cell Hub 2 (TISCH2) was used as a resource to analyze TNFAIP3 gene expression levels in individual cell populations of 7 independent single-cell RNA-seq (scRNA-seq) HNSCC patient cohorts (https://pubmed.ncbi.nlm.nih.gov/36321662/, last accessed on 7 January 2023). Results of these publicly-available scRNA-seq data are displayed as Supplementary Figure S1.

## 3. Results

**Figure 1:**
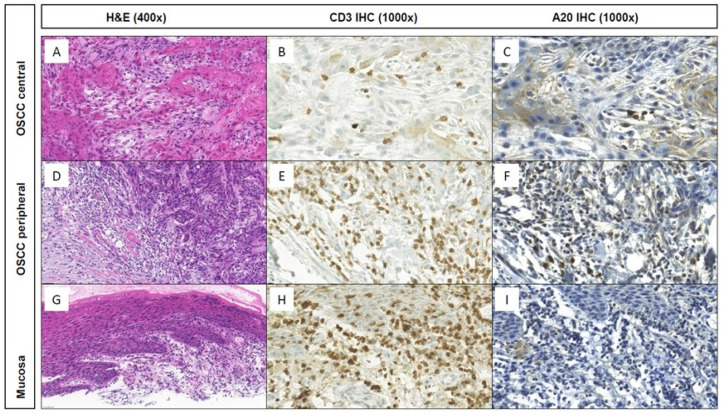
Histomorphology (H&E; (**A**,**D**,**G**)) and CD3 and A20 IHC stainings at central and peripheral tumor areas and the associated non-neoplastic mucosa of a representative keratinizing OSCC case of the TMA cohort. Whereas only few or no stromal CD3+ T cells within the tumor center (**B**,**C**) and non-neoplastic mucosa (**H**,**I**), respectively, show A20 IHC expression, high A20 expression was observed within the stromal CD3+ T cells at the tumor periphery (**E**,**F**).

**Figure 2:**
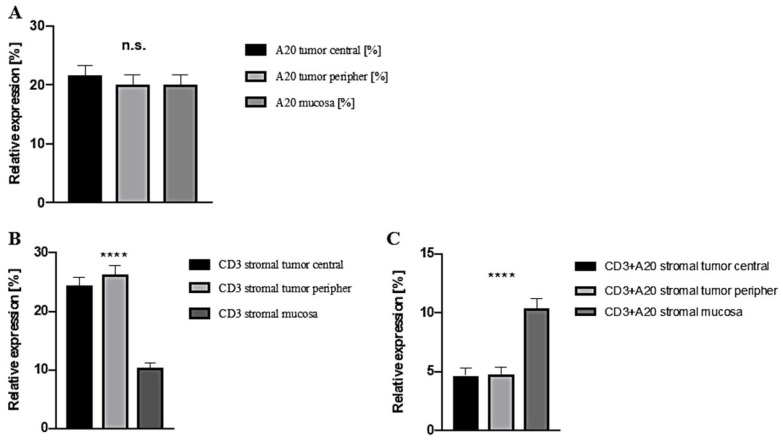
(**A**) Analysis of relative A20 IHC expression in central and peripheral OSCC as well as non-malignant mucosa (*n* = 172); (**B**) Expression of stromal CD3+ T cells in the tumor center, the tumor periphery and non-malignant mucosa; (**C**) A20 IHC expression within stromal CD3+ T cells in tumor center, tumor periphery as well as non-malignant mucosa; **** *p* < 0.0001, n.s. = not significant.

Before we analyzed the present OSCC TMA in view of A20 expression, we questioned which cell types might exceed in A20 expression. Hereby, we characterized A20 expression in the tumor microenvironment, based on publicly available scRNA-seq data of seven independent HNSCC cohorts. In this regard, we were able to show that TNFAIP3 gene expression levels (codes for A20) are mainly detected in immune cell populations and hereby specifically in T cell subsets (Supplementary Figure S1). Within our retrospective cohort study, an OSCC TMA was analyzed according to A20 expression in three different localizations: The central tumor, peripheral tumor parts as well as non-malignant oral mucosa (Figure 1). IHC was assessed for CD3 as well as A20 besides standard H&E-staining. The quantitative analysis of A20 IHC showed a quite similar expression in central and peripheral malignant areas as well as in non-malignant oral mucosa (Figure 2A). H&E and CD3 IHC stainings revealed a high stromal infiltration of T cells in central and peripheral tumor parts when compared to normal mucosa (Figure 2B). Within stromal CD3+ T cells, peripheral as well as central tumor regions could be identified to poorly express A20, whereas normal mucosa presented significantly more A20+ T cells (Figure 2C). Based on these findings, we henceforth concentrated on A20 expression on stromal CD3+ T cells in the tumor periphery. For this subcohort, 172 patients could be analyzed in detail (Table 1), for the remaining 57 patients, at least one of the three tissues did not meet quality requirements to get included in this study. Thereby, 123 patients were male (71.5%), the mean age was 60.5 years (61.3 years in the group of A20-overexpression), and additionally, most patients had a positive alcohol (71.5%) or smoking anamnesis (80.2%). Predominant tumor localization was the floor of the mouth (47.1%) and the lower alveolus and gingiva (21.5%). The majority of tumors was staged as pT2 (39.5%) and pT4 (30.2%), 57.7% of included patients had no cervical lymph node metastasis (pN0), however, 48.18% of cases were already staged as UICC-stage IV. Therefore, the majority of patients received adjuvant therapy modalities, hereby, 73 patients (42.4%) were treated with radiotherapy (RT) whereas 27 cases received adjuvant radiochemotherapy (RCT; 15.7%). Using the median A20 expression within stromal CD3+ T cells (2.5%) as the cut-off, the correlation of high A20 expression with clinicopathological characteristics is also presented in Table 1.

The Chi-square test revealed no significant correlation of A20 overexpression with clinical as well as pathohistological characteristics of OSCC patients (Table 1).

**Figure 3:**
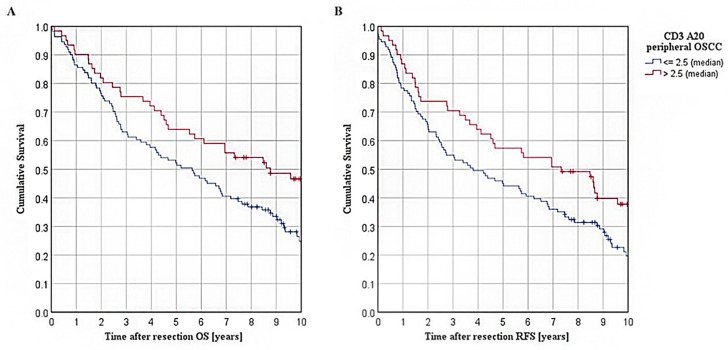
Survival in TMA cohort of OSCC patients: (**A**): Kaplan–Meier curves for OS in patients with varying extent of A20 expression within stromal CD3+ T cells of peripheral tumor, separated by a median cut-off (*n* = 172, *p* = 0.017); (**B**): Kaplan–Meier curves for RFS in patients with varying extent of A20 expression within stromal CD3+ T cells of peripheral tumor, separated by a median cut-off (*p* = 0.020).

Furthermore, survival in OSCC patients was analyzed according to A20 expression within stromal CD3+ T cells in the peripheral tumor area. Hereby, Kaplan–Meier curves are shown in Figure 3A,B. For the patient collective with an A20 overexpression (A20 > median), the Kaplan–Meier analysis revealed a significantly ameliorated five-year OS of 63.9% when compared to patients with a low A20 expression within peripheral stromal CD3+ T cells (52.3% five-year OS for A20 ≤ median; *p* = 0.017; Figure 3A). For RFS, similar results were observed in both groups (57.4% five-year RFS for A20-high, 45.0% five-year RFS for A20-low; *p* = 0.020; Figure 3B).

Subsequently, we performed uni- and multivariable survival analyses to adjust for covariables. For OS, the multivariable Cox regression analysis revealed elevated comorbidities, reflected by the age-adjusted CCI, to significantly impair the survival of OSCC patients (for ACCI > 4: HR 2.600; 95% CI 1.539–4.395, *p* < 0.001) (Table 2). UICC-stage thereby missed statistical significance, however, a clear trend could still be observed for advanced UICC-stages (III & IV) to impair the OS of tumor patients (HR 1.486; 95% CI 0.996–2.217, *p* = 0.052). In contrast, A20 overexpression within stromal CD3+ T cells in the peripheral tumor area was significantly associated with ameliorated OS of tumor patients in the multivariable survival analysis (0.582; 95% CI 0.388–0.873, *p* = 0.009). Comparable results were observed in multivariable Cox-regression for RFS (for ACCI > 4: HR 2.025; 95% CI 1.226–3.346, *p* = 0.006) (A20 high: HR 0.605; 95% CI 0.411–0.889, *p* = 0.011) (Table 3).

## 4. Discussion

NF-κB regulating protein A20 plays an important role in several autoimmune and inflammatory diseases, furthermore, it acts as a tumor suppressor protein in different hematological neoplasms such as B cell lymphoma [17]. In this context, a genome-wide analysis was performed and A20 was shown to be inactivated due to somatic mutations or deletions in various kinds of B-lineage lymphoma such as Hodgkin’s lymphoma [17]. These observations directly implicated a potential impact of A20 also in other tumor entities. In solid tumors such as colorectal cancer or hepatocellular carcinoma for instance, A20 was identified as a potent tumor suppressor [20,28]. However, when it comes to the prognostic role of A20 in solid tumors, the current literature is rather inconclusive; according to Shi et al., the prognostic role of A20 in malignant diseases should be revealed for every entity individually as the impact varies between different tumor types [29]. In gastric and breast cancer as well as in malignant melanoma, strong oncogenic properties have been attributed to A20, which was not the case in colorectal cancer or hepatocellular carcinoma [22,30,31]. To gain deeper insight into the impact of A20 on malignant diseases, its role in tumorigenesis and tumor progression has to be investigated in detail. In this context, the observed tumor-suppressive effect of A20 was repeatedly investigated by various authors. In terms of possible underlying mechanisms of how A20 entails anti-tumor properties, inhibition of NF-κB signaling has frequently been proposed [29]. In different malignant diseases, the NF-κB pathway was shown to be strongly activated [32], thereby inducing tumor cell proliferation, apoptosis resistance and ultimately distant disease manifestation directed by epithelial to mesenchymal transition (EMT) [32]. In this regard, it has been shown for A20 that it was able to control NF-κB signaling in vitro and thereby facilitating induction of tumor cell apoptosis as well as growth arrest in lymphoma cell lines [33].

In contrast to these anti-tumorigenic properties, however, A20 is also involved in various pro-tumorigenic mechanisms. In breast cancer, A20 was shown to favor metastasis formation via monoubiquitylation of Snail1 [34]. These differential findings were recently brought together in a publication by Shi et al., demonstrating “the dual roles of A20 in cancer” [29]. In this context, the authors favor a “cancer type-dependent” approach when further evaluating the role of A20 in different malignancies [29]. Even though comprehensive research has been conducted in determining the role of A20 in the tumorigenesis and progression of distinct cancer types, the tumor entity OSCC has not been adequately assessed. Concerning this, we were not only able to show, that A20 is remarkably downregulated in CD3+ cells of OSCC compared to non-malignant oral mucosa. When comparing these results with the current literature, little is known about A20 expression in tumors of the head neck region. However, Codd et al. published the results of A20 in nasopharyngeal tumors as well as in poorly differentiated head and neck squamous cell carcinoma, demonstrating that a more undifferentiated cancer phenotype is directly linked with elevated A20 expression [35]. Based on these findings and in view of more recent publications, revealing A20 as a negative parameter on the outcome of breast cancer patients [21], results of the present cohort study certainly require a deeper insight in possible underlying mechanisms.

In this regard, A20 has recently been regarded from a different point of view: Guo et al. showed insights in tumor immunology of malignant melanoma, pointing out the strongly relevant role of A20 in the anti-cancer immune response [36]. Especially when it comes to establishing novel strategies for patients, refractory to programmed cell death 1 (PD-1) antibody treatment, A20 was identified as a promising target to enhance patients’ responses towards immune checkpoint therapies [36]. Especially after the broad application of anti-PD-1/anti- programmed cell death 1 ligand 1 (PD-L1) immune therapy in tumors of the head and neck and oral cavity [37,38], overcoming therapy resistance is one of the major challenges of translational research in OSCC [37].

A limitation of the present study is certainly the retrospective approach that needs to be further evaluated in consecutive, prospective clinical trials. Our study is based on IHC stainings of distinct parts of tumor tissue as well as corresponding non-malignant mucosa, which—due to protein-based evaluation within the tumor samples—allows a detailed insight in A20 expression in OSCC. Especially in combination with extensive clinicopathological characteristics as well as survival data, we are firmly convinced, that this work contributes significantly to understanding the role of A20 as a novel prognostic marker in OSCC.

## 5. Conclusions

The present work characterizes A20 as a novel prognostic biomarker in OSCC. A20 overexpression within stromal CD3+ T cells leads to the significantly improved survival of cancer patients. Especially due to the predominant expression of A20 on stromal tumor infiltrating CD3+ T cells, further research, especially in regards to establishing innovative treatment concepts such as immune checkpoint therapies, is highly needed.

## Figures and Tables

**Table 1:** Patients’ characteristics according to A20 expression within stromal CD3+ T cells at tumor periphery (*n* = 172).

		**A20 Expression within Stromal CD3+ T Cells at A20 Stromal Peripheral Tumor Periphery**						
		A20-low		A20-high		Total		*X^2^*
		Count	%	Count	%	Count	%	*p*
Sex	Female	29	26.1%	20	32.8%	49	28.5%	0.354
	Male	82	73.9%	41	67.2%	123	71.5%	
Age at diagnosis	<50	20	18.0%	10	16.4%	30	17.4%	0.352
	50.0–59.9	42	37.8%	17	27.9%	59	34.3%	
	60.0–69.9	24	21.6%	22	36.1%	46	26.7%	
	70.0–79.9	19	17.1%	9	14.8%	28	16.3%	
	≥80.0	6	5.4%	3	4.9%	9	5.2%	
CCI age adjusted	0–1	32	28.8%	19	31.1%	51	29.7%	0.731
	2	21	18.9%	14	23.0%	35	20.3%	
	3	23	20.7%	10	16.4%	33	19.2%	
	4	12	10.8%	9	14.8%	21	12.2%	
	≥5	23	20.7%	9	14.8%	32	18.6%	
Positive smoking anamnesis	No	20	18.0%	14	23.0%	34	19.8%	0.437
	Yes	91	82.0%	47	77.0%	138	80.2%	
Positive alcohol anamnesis	No	30	27.0%	19	31.1%	49	28.5%	0.567
	Yes	81	73.0%	42	68.9%	123	71.5%	
Anatomical site	Buccal mucosa	14	12.6%	7	11.5%	21	12.2%	0.798
	Upper alveolus and gingiva	7	6.3%	1	1.6%	8	4.7%	
	Lower alveolus and gingiva	23	20.7%	14	23.0%	37	21.5%	
	Hard palate	4	3.6%	2	3.3%	6	3.5%	
	Tongue	11	9.9%	8	13.1%	19	11.0%	
	Floor of mouth	52	46.8%	29	47.5%	81	47.1%	
Tumor size	T1	24	21.6%	17	27.9%	41	23.8%	0.164
	T2	40	36.0%	28	45.9%	68	39.5%	
	T3	7	6.3%	4	6.6%	11	6.4%	
	T4	40	36.0%	12	19.7%	52	30.2%	
Cervical node status	N0	55	49.5%	34	55.7%	89	51.7%	0.223
	N1	17	15.3%	13	21.3%	30	17.4%	
	N2/3	39	35.1%	14	23.0%	53	30.8%	
Extranodal spread	No	42	37.8%	22	36.1%	64	37.2%	0.601
	Yes	14	12.6%	5	8.2%	19	11.0%	
	not applicable	55	49.5%	34	55.7%	89	51.7%	
Tumor grade	G1	5	4.5%	3	4.9%	8	4.7%	0.992
	G2	93	83.8%	51	83.6%	144	83.7%	
	G3/4	13	11.7%	7	11.5%	20	11.6%	
Lymph vessel invasion	L0	87	78.4%	51	83.6%	138	80.2%	0.410
	L1	24	21.6%	10	16.4%	34	19.8%	
Blood vessel invasion	V0	104	93.7%	59	96.7%	163	94.8%	0.394
	V1	7	6.3%	2	3.3%	9	5.2%	
UICC stage	I	17	15.3%	11	18.0%	28	16.3%	0.666
	II	21	18.9%	13	21.3%	34	19.8%	
	III	15	13.5%	11	18.0%	26	15.1%	
	IV	58	52.3%	26	42.6%	84	48.8%	
Adjuvant therapy	No	48	43.2%	24	39.3%	72	41.9%	0.570
	Radiotherapy	44	39.6%	29	47.5%	73	42.4%	
	Radiochemotherapy	19	17.1%	8	13.1%	27	15.7%	
Recurrence	No recurrence	79	71.2%	47	77.0%	126	73.3%	0.405
	Recurrence	32	28.8%	14	23.0%	46	26.7%	
Death	Alive	30	27.0%	22	36.1%	52	30.2%	0.217
	Total	111	100.0%	61	100.0%	172	100.0%	

**Table 2:** Results from univariable and multivariable Cox-regression analyses for overall survival in patients according to high and low A20 expression, respectively (cut-off: 2.5 (median value)) within stromal CD3+ T cells in the peripheral tumor area. Multivariable analysis adjusted for age-adjusted Charlson comorbidity score, UICC stage, lymph vessel and blood vessel invasion, which proved to have *p* < 0.100 in univariable analysis. Tumor size, cervical node status and extranodal spread were dismissed in favor of UICC stage.

		Univariable Cox-Regression				Multivariable Cox-Regression			
				95%-CI				95%-CI	
		*p*	HR	Lower	Upper	*p*	HR	Lower	Upper
CD3+ A20 stromal peripheral	A20-low								
	A20-high	0.018	0.620	0.417	0.920	0.009	0.582	0.388	0.873
Sex	Female								
	Male	0.876	1.033	0.689	1.549				
CCI age adjusted	0–1	<0.001				<0.001			
	2	0.319	1.327	0.760	2.317	0.237	1.408	0.798	2.485
	3	0.651	1.143	0.641	2.040	0.768	1.091	0.611	1.950
	4	0.001	2.645	1.455	4.807	<0.001	3.115	1.696	5.721
	≥5	<0.001	2.583	1.551	4.302	<0.001	2.600	1.539	4.395
Positive smoking anamnesis	No								
	Yes	0.809	0.945	0.598	1.494				
Positive alcohol anamnesis	No								
	Yes	0.667	1.093	0.729	1.640				
Anatomical site	Upper alveolus and gingiva and hard palate	0.388							
	Tongue	0.196	0.558	0.230	1.352				
	Buccal mucosa and lower alveolus and gingiva and floor of mouth	0.560	0.816	0.411	1.618				
UICC stage	I and II								
	III and IV	0.015	1.610	1.095	2.368	0.052	1.486	0.996	2.217
Tumor grade	G1/2								
	G3/4	0.143	1.484	0.875	2.517				
Lymph vessel invasion	L0								
	L1	0.046	1.559	1.008	2.412	0.163	1.417	0.869	2.313
Blood vessel invasion	V0								
	V1	0.023	2.313	1.121	4.775	0.347	1.475	0.656	3.320
Adjuvant/additive therapy	No	0.263							
	Radiotherapy	0.123	1.363	0.920	2.021				
	Radiochemotherapy	0.266	1.357	0.793	2.324				

**Table 3:** Results from univariable and multivariable Cox-regression analyses for recurrence-free survival in patients according to high and low A20 expression, respectively (cut-off: 2.5 (median value)) within stromal CD3+ T cells in the peripheral tumor area. Multivariable analysis adjusted for age-adjusted Charlson comorbidity score, UICC stage, vein invasion and adjuvant/additive radiotherapy, which proved to have *p* < 0.100 in univariable analysis. Tumor size, cervical node status and extranodal spread were dismissed in favor of UICC stage.

		Univariable Cox-Regression				Multivariable Cox-Regression			
				95%-CI				95%-CI	
		*p*	HR	Lower	Upper	*p*	HR	Lower	Upper
CD3+ A20 stromal peripheral	A20-low								
	A20-high	0.021	0.643	0.441	0.936	0.011	0.605	0.411	0.889
Sex	Female								
	Male	0.509	1.143	0.769	1.698				
CCI age adjusted	0–1	0.005				0.002			
	2	0.588	1.157	0.682	1.962	0.648	1.132	0.665	1.928
	3	0.612	1.150	0.670	1.974	0.718	1.105	0.642	1.902
	4	0.002	2.443	1.374	4.346	0.001	2.619	1.464	4.684
	≥5	0.006	1.993	1.217	3.265	0.006	2.025	1.226	3.346
Positive smoking anamnesis	No								
	Yes	0.836	0.954	0.611	1.489				
Positive alcohol anamnesis	No								
	Yes	0.804	0.952	0.648	1.401				
Anatomical site	Upper alveolus and gingiva and hard palate	0.296							
	Tongue	0.242	0.590	0.244	1.427				
	Buccal mucosa and lower alveolus and gingiva and floor of mouth	0.911	0.962	0.486	1.902				
UICC stage	I and II								
	III and IV	0.015	1.587	1.095	2.301	0.207	1.317	0.859	2.021
Tumor grade	G1/2								
	G3/4	0.102	1.535	0.919	2.563				
Lymph vessel invasion	L0								
	L1	0.128	1.392	0.909	2.129				
Blood vessel invasion	V0								
	V1	0.083	1.894	0.920	3.898	0.362	1.425	0.666	3.047
Adjuvant/additive therapy	No	0.097				0.297			
	Radiotherapy	0.060	1.441	0.985	2.109	0.149	1.379	0.891	2.134
	Radiochemotherapy	0.083	1.577	0.943	2.639	0.211	1.435	0.815	2.525

## Data Availability

Data can be obtained by scientists that work independently from the industry on request. Data are not stored on publicly available servers.

## Supplementary Materials

The following supporting information can be downloaded at: https://www.mdpi.com/article/10.3390/cancers15030675/s1, Figure S1: Average TNFAIP3 gene expression levels in individual cell types of 7 independent HNSCC patient cohorts which were analyzed by single cell RNA-sequencing.
