# Supplementary material for: A20 as a Potential New Tool in Predicting Recurrence and Patient’s Survival in Oral Squamous Cell Carcinoma
Source: Cancers (Basel). 2023 Jan 21;15(3):675. doi: 10.3390/cancers15030675 (PMC9913673; doi:10.3390/cancers15030675)
Supplement: Supplementary file 1 [file cancers-15-00675-s001.zip › cancers-2147204-supplementary.pdf]

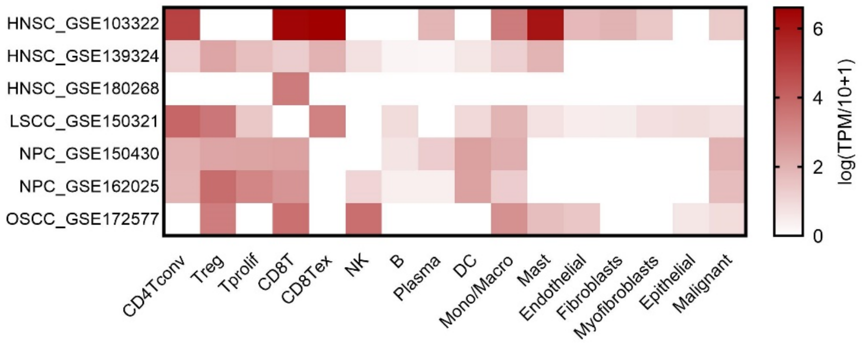

Supplementary Figure S1. Average TNFAIP3 gene expression levels in individual cell types of 7 independent HNSCC patient cohorts which were analyzed by single cell RNA-sequencing.
